# HOME HEALTH CARE USE AND OUTCOMES AMONG PERSONS WITH DEMENTIA: A SCOPING REVIEW

**DOI:** 10.1093/geroni/igae098.2062

**Published:** 2024-12-31

**Authors:** Jamie Smith, Julia Burgdorf, Tiffany Riser, Miriam Ryvicker

**Affiliations:** Widener University, Chester, Pennsylvania, United States; VNS Health, New York, New York, United States; Johns Hopkins University, Baltimore, Maryland, United States; VNS Health, New York, New York, United States

## Abstract

One-third of home healthcare (HHC) recipients are persons living with dementia (PLWD). HHC provides skilled nursing and therapy delivered in the patient’s home. Amid population aging and a shift towards home- and community-based care, there is a growing need for HHC services for PLWD. However, dementia care research has historically focused on institutional care settings, and HHC has been understudied. We conducted a scoping review to summarize the current research landscape regarding HHC utilization and clinical outcomes for PLWD and recommend future research directions. We applied Arksey & O’Malley’s methodology and identified 32 articles that met inclusion criteria. Of these, 22 studies examined utilization, finding that PLWD used HHC differently than persons without dementia, having a higher likelihood of community referral, longer episode length, greater use of nursing and aide services, and increased costs. Sixteen papers reported on clinical outcomes: function, hospitalization, skilled nursing facility admission, and mortality. Analyses varied widely in their approach to defining dementia and measuring key outcomes. However, literature suggests an increased risk for negative outcomes, including hospitalization and institutionalization among PLWD. Findings highlight several areas for future research, most saliently, the need for analyses that consider contextual factors (e.g., market factors, racial/ethnic disparities, rurality) when examining HHC for PLWD. Future work should assess the impacts of payment model revisions and expanding Medicare Advantage coverage on utilization and care delivery for this population. A rigorous examination of care delivery and outcomes is needed to inform practice models to adapt to the unique care needs of PLWD.

